# History of Neurotrauma: A tale from the Medieval Ages and the Renaissance

**DOI:** 10.25122/jml-2023-1001

**Published:** 2023-01

**Authors:** Stefana-Andrada Dobran, Dafin Muresanu

**Affiliations:** 1 RoNeuro Institute for Neurological Research and Diagnostic, Cluj-Napoca, Romania; 2 Sociology Department, Babes-Bolyai University, Cluj-Napoca, Romania; 3 Department of Neuroscience, Iuliu Hatieganu University of Medicine and Pharmacy, Cluj-Napoca, Romania

## NEUROTRAUMA DURING THE DARK AGES: THE STORY OF BERENGARIO DA CARPI

During the medieval period, the scientific approach to medicine advocated by Hippocrates was overshadowed by a belief in magic and mysticism [1]. Europe entered the Dark Ages, where medical practices relied on prayers and spells instead of judgment and reason. Church clerks and monks replaced physicians, and illnesses were attributed to sin and witchcraft. The Renaissance marked a shift away from this supernatural approach to medicine towards a more empirical and critical observation [1].

**Figure 1:**
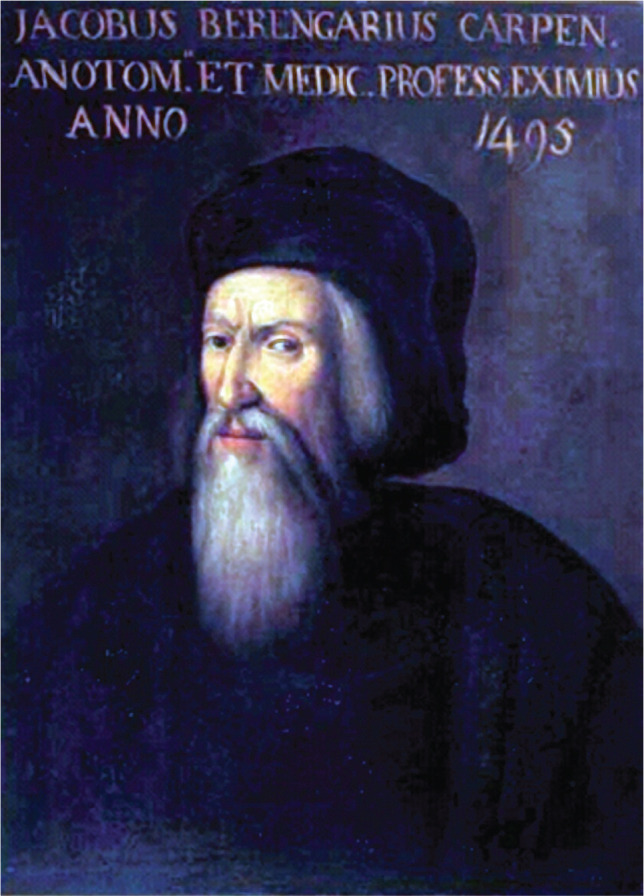
Portrait of Berengario da Carpi [2].

Jacopo Berengario da Carpi was a physician famous for his contribution to the field of neurotrauma during medieval times (circa 1460) (Figure 1) and a true “Renaissance man” [2]. He received a degree in philosophy and medicine from the University of Bologna in northern Italy and became a lecturer in anatomy and surgery at age 40 [2,3]. As an anatomist and surgeon, he published “*Commentaria cum amplissimis additionibus super Anatomia Mundini*” in 1521, the first illustrated anatomic textbook ever printed [3].

Another significant work of Berengario is “*Tractatus de Fractura Calvae sive Cranei*”, published in 1518. It is the first comprehensive treatise on head injuries and the most notable work on craniocerebral surgery from the 16^th^ century [2,3]. This book represents a milestone in cranial surgery, presenting case reports of neurotrauma fatalities, including depictions of “subdural and epidural hematomas, contrecoup lesions, and subdural empyema” from patients who died of head trauma [2]. Berengario accomplished this impressive effort in just two months and dedicated the book to Lorenzo de Medici, Duke of Urbino, after he sustained an occipital skull injury [2,3].

In his work, Berengario introduced the world's first illustrated surgical kit for cranial surgeries, known as "corpus instrumentorum," and explained the craniotomy procedure in detail [3]. The modernity of his works is striking, as it deals with the mechanisms, diagnostics, and treatments of head injury. “De Fractura Calvae sive Craniei” mentions causes and types of skull fractures (a) intrinsic and (b) extrinsic (cut, contusion, perforation), descriptions of symptoms of a variety of injuries (dura lesions, cerebral contusions, vessel injuries, brain hemorrhage, and brain hematoma) which include vomiting, loss of speech, vertigo, and loss of equilibrium. Furthermore, the author describes the prognosis of head trauma, advises not to underestimate neurotrauma, and presents possible therapies (particularly craniotomy in the temporal lobe). In addition, he presents various instruments for surgery, such as trephines, elevators, forceps, and chisels [3].

Berengario made significant contributions to the field of neurotrauma. He treated many well-known figures of the time, including several members of the Pope’s family, and also became known for using mercury to treat syphilis [2]. Berengario also placed emphasis on the importance of pulse and facial expression, possibly recognizing anisocoria, offered significant importance to convalescence and nutrition, and considered the psychological effects of neurotrauma by advising patients to avoid negative emotions (anger, fear, sadness, and fury post-injury) [2]. With a deep dedication to his work, Berengario taught his students that “*a physician is not a carpenter but works with the human body [...]. If imperfect handling occurs in a human being, the individual is destroyed and cannot ever be restored*” [2].

## NEUROTRAUMA – PERSPECTIVES FROM EUROPE

**Figure 2:**
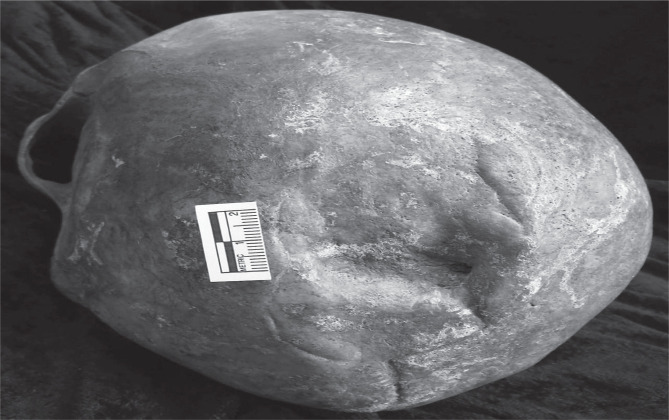
A massive depression fracture on the left parietal of a Sortebrødre cranium and a radiating fracture extending to the occipital bone [4].

The significance of head trauma in anthropology is also evidenced in medieval Denmark, where skeletal observations showed a high risk for male neurotrauma, occurring more often than female neurotrauma, representative of a high rate of interpersonal violence (Figure 2) [4]. The impact of neurotrauma was considerable in those times, as even minor injuries could pose a significant risk and an increased risk for mortality, especially during economic hardships [4].

The 13^th^ century saw the emergence of a remarkable figure in the field of neurotrauma, Guido Lanfranchi [5]. He discussed concussion as a separate entity and pinpointed that the disappearance of symptoms post-injury resulted from transient paralysis caused by the brain being shaken, highlighting the concept of commotion, which was vital in the later physiological understanding of concussion [5]. Another notable figure in the field of neurotrauma was Hieronymus Brunschwig, a German surgeon who published the first work on gunshot wounds and described penetrating head wounds and skull fractures. Ambroise Paré, an emblematic barber-surgeon of the Renaissance, described, in his works, commotions or “commotio” as disorders of brain movement, with swelling and hemorrhage, putting the term into actuality. Paré made recommendations for examining the overlying scalp to better examine the bone. The distinction between cerebral commotion and contusion began with Lanfrancus and Berengario da Carpi [5,6].

Surgeons were more often seen as “artisans” rather than scientists, yet they played a tremendously vital role in the advancement of science [6]. Moreover, although previously used for rather spiritual purposes, the trepanation procedure was reintroduced for severe head injuries [6].

Other notable figures of the time were Dino del Garbo, who promoted the use of plasters rather than surgical procedures for the treatment of head injuries along with the differentiation of skull fractures from other head wounds, and Francisco Arceo, a Spanish military surgeon who wrote remarkable works regarding head trauma [6].

Thomas Sydenham, an English physician of the 17^th^ century, was recognized for his approach to medicine, which relied on observation, rational examination, and intellectual speculation. He was known as the “English Hippocrates” and the father of clinical observations for incorporating the role of environmental factors, stress, diet, exercise, and emotions into the clinical assessment process. He believed in following the rational method of medicine, inspired by the teachings of Hippocrates [1].

## NEUROTRAUMA THROUGHOUT THE CENTURIES

The spirit of the Renaissance was one of curiosity, innovation, and revived fascination for science, and the period highlighted a renewed interest in Greek and Roman works [6]. Throughout the centuries, the management of neurotrauma, both in terms of evolution and understanding, has been marked by striking changes. From ancient times to the Renaissance, brilliant surgeons, anatomists, and physicians significantly contributed to advancing the field. From recognizing the importance of pulse and facial expression to exploring the use of trepanation and plasters to advocating for clinical observations, the history of neurotrauma has been shaped by a spirit of curiosity, innovation, and a relentless drive toward improving patient outcomes. Today, neurotrauma continues to be a complex and challenging field, requiring a multidisciplinary approach. However, the foundation of knowledge and skills developed over centuries provides a solid basis for future advancements.

## References

[1] Stiefel M, Shaner A, Schaefer SD (2006). The Edwin Smith Papyrus: the birth of analytical thinking in medicine and otolaryngology. Laryngoscope.

[2] Di Ieva A, Gaetani P, Matula C, Sherif C (2010). Berengario Da Carpi: A Pioneer in Neurotraumatology-Historical Vignette. Journal of Neurosurgery.

[3] Mazzola R, Mazzola IC (2009). Treatise on Skull Fractures by Berengario Da Carpi (1460-1530). Journal of Craniofacial Surgery.

[4] Boldsen JL, Milner GR, Weise S (2015). Cranial Vault Trauma and Selective Mortality in Medieval to Early Modern Denmark. Proceedings of the National Academy of Sciences of the United States of America.

[5] McCrory PR, Berkovic SF (2001). Concussion: The history of clinical and pathophysiological concepts and misconceptions. Neurology.

[6] Nathan B, Evans G (1998). The Treatment of Head Injury during the Renaissance. Journal of Accident & Emergency Medicine.

